# A Psychoanalytical Perspective on the Co-therapeutic Relationship With a Group of Siblings of Children With Autism: An Observational Study of Communicative Behavior Patterns

**DOI:** 10.3389/fpsyg.2019.01832

**Published:** 2019-08-08

**Authors:** Mariella Venturella, Xavier Carbonell, Víctor Cabré, Eulàlia Arias-Pujol

**Affiliations:** ^1^FPCEE Blanquerna, Ramon Llull University, Barcelona, Spain; ^2^Training and Research Center, Carrilet, Barcelona, Spain

**Keywords:** co-therapy, siblings, autism spectrum disorder, group psychotherapy, therapeutic communication, mixed method, systematic observation

## Abstract

A child diagnosed with autism may have a negative psychological and behavioral impact on their siblings, whose participation in a group with children in the same situation is a preventive measure. Our group study was conducted by two therapists (T1 and T2) assigned to co-therapy (CT) work. Both therapists shared the theoretical bases and understanding of the group and the needs of the individual subjects, and complemented each other in terms of the direction of their interventions, given that shared impressions and continuous exchanges that integrate countertransference aspects are essential to successful co-therapy. The objectives of this study were as follows: (a) to detect patterns of clarification, confrontation, and interpretation interventions by T1 and T2 in the group; and (b) to detect patterns of clarification, confrontation and interpretation interventions considering T1 and T2 as the only focal subject of the CT. Design was mixed-methods based on systematic observation, for which we developed a qualitative *ad hoc* instrument that combined a field format and a categorizing system. Interobserver agreement was analyzed quantitatively using Cohen’s kappa and Krippendorf’s canonical concordance. Once data reliability was confirmed, lag sequential analysis using GSEQ5 software was performed to search for behavior patterns. The results show (a) different behavior patterns in the clarification, confrontation, and interpretation interventions by T1 and T2; and (b) different behavior patterns when T1 and T2 are considered as the focal subject (CT). Our study offers a new perspective on the impact of therapist interventions on participants in this kind of group.

## Introduction

Family centered care in the autism spectrum disorder (ASD) field has attracted growing interest in the past decade in early care units (Gabovitch and Curtin, 2009; Christon and Myers, 2015). Studies focused on children with siblings with ASD (ASD-Sibs) have adopted different approaches. Hypothesizing genetic vulnerability (Cassel et al., 2007), a number of follow-up studies have sought to identify early stage ASD-Sibs predictors (Yoder et al., 2009). Another perspective has sought to identify the benefits of the sibling bond for autistic children, with a retrospective study reporting that older siblings positively influence the social skills of younger ASD-Sibs (Ben-Itzchak et al., 2018). In recent years, interest has grown in whether ASD-Sibs have a greater risk of developing emotional and behavioral problems than the general population, with empirical results pointing to enormous variability (Griffith et al., 2014; McHale et al., 2016): some studies affirm an increased risk (Meyer et al., 2011; Shivers et al., 2013; Hastings and Petalas, 2014), others suggest a similar risk (Macks and Reeve, 2007; Ferraioli and Harris, 2009; Walton and Ingersoll, 2015), and yet others argue that ASD-Sibs demonstrate better social adaptation and more positive sibling relationships (Hastings, 2003; Petalas et al., 2012). A recent meta-analysis (Shivers et al., 2018) of 69 studies that compared siblings with ASD-Sibs with siblings without ASD-Sibs found that, for some 800 individual comparisons, children with ASD-Sibs had significantly poorer – albeit small in magnitude – outcomes, specifically in their internalization of behavioral problems, psychological functioning, beliefs, social functioning, and relationships between siblings. No significant results were obtained for adaptation, externalization of behavioral problems, attention-deficit/hyperactivity disorder, coping or family functioning.

Support groups for ASD-Sibs are key to the prevention and early detection of developmental and emotional disorders and also in terms of therapeutic strategies when difficulties appear (Shivers et al., 2018). Traditionally, interventions for ASD-Sibs are implemented through support groups for family members, focused on communicative strategies that foster positive relations between siblings and a climate of trust in the family that facilitates the revelation of thoughts and feelings (Harris and Glasberg, 2003). Programs are available that seek to empower ASD-Sibs to stimulate and play with their siblings with autism and help them acquire social skills (Tsao and McCabe, 2010; Tsao et al., 2012). Since the behavioral problems frequently associated with ASD may lead to the emergence of negative emotions in the siblings (Tsao et al., 2012), some studies have underlined the need for siblings to be able to express feelings and thoughts (Mascha and Boucher, 2006; Angell et al., 2012), although – as if they recognize that there is no room in the family for further problems – ASD-Sibs often appear to have no difficulty in adapting and are understanding and responsible. However, it has also been observed that these children may deliberately hide their need for a space to be someone other than “the brother or sister of” their sibling (Centre Educatiu i Terapèutic Carrilet, Alcácer et al., 2013; Farrés, 2014).

**FIGURE 1:**
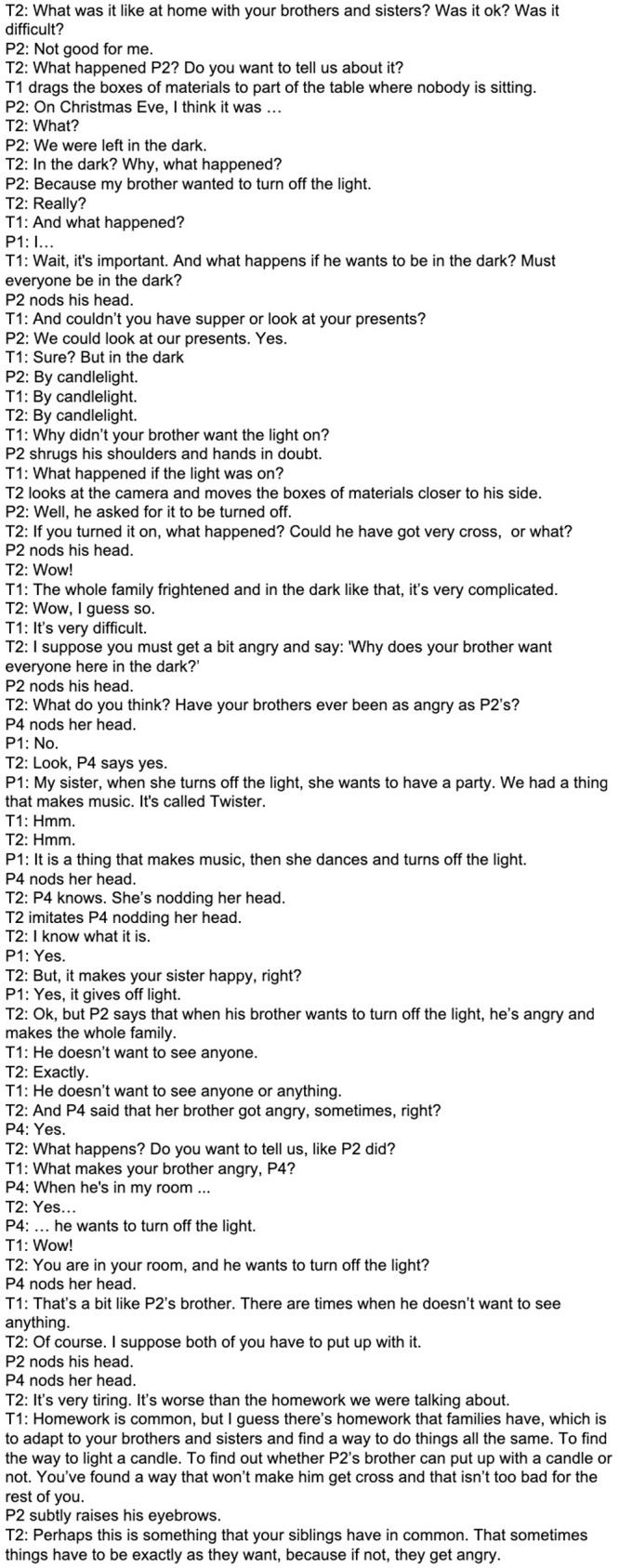
Fourth group session transcriptions.

The goal of our sibling support group is to offer a space where ASD-Sibs can freely express feelings and thoughts that may remain silenced in their everyday life or that may be perceived as contradictory. In the group the children explore the ambivalence of wanting to care for their sibling with special needs while also feeling guilt, anger and maybe even hate because of the special treatment their sibling receives from parents or at school. Figure 1 shows, as an example, an excerpt from a session in which siblings are discussed (Centre Educatiu i Terapèutic Carrilet, Alcácer et al., 2013; Farrés, 2014).

In this research, we wanted to study the spontaneous interaction between children and psychoanalytical therapists in a group. This required a flexible methodology adaptable to all behaviors and contexts. Mixed methods research offers an excellent combination of rigor and flexibility while allowing qualitative and quantitative techniques to be used within the same paradigm (Creswell et al., 2003; Johnson et al., 2007; Tashakkori and Teddlie(eds), 2010). Indeed, qualitative and quantitative techniques are gradually progressing toward integration (Hesse-Biber and Johnson, 2013; Fetters and Freshwater, 2015; Onwuegbuzie et al., 2018), although some problems remain, such as how to achieve that integration (Bryman, 2007; Bazeley, 2012, 2017) and its translation into practice (Onwuegbuzie and Leech, 2005; Happ et al., 2006). Mixed methods, which involve combining inductive logic with deductive logic (Bergman, 2010) throughout the entire research process, has an integral role to play in “complete” methodological development. Observational methodologies have been pioneering in achieving this methodological complementarity (Anguera et al., 2001; Anguera and Izquierdo, 2006; Sánchez-Algarra and Anguera, 2013), e.g., the recent conceptualization in indirect observation (Anguera et al., 2018).

The conceptual framework for this study gave rise to four essential dimensions reflected in the observation instrument: ASD-Sib, turn-taking, group, and play. However, although all the small children in the support group shared the fact of having a sibling with ASD, there were few explicit interventions about the sibling. Group activities focused on plasticine models and wooden doll families. The plasticine figures made by the children were kept, which led to some becoming characters in the group (Venturella et al., 2015).

In this mixed-methods study we focus on the group and turn-taking dimensions of the therapists. From a psychoanalytical theory perspective, the group interventions of the therapists were divided into clarification (I1), confrontation (I2), and interpretation (I3). Clarification allows the therapist to emphasize essential elements in communications and perceptions, confrontation stimulates an interest in thinking, reflecting, and understanding behavior in relation to others, and interpretation is a hypothesis as to how a group participant may feel in the here and now of a session (Coderch, 2009; Ferro and Civitarese, 2016).

In this study we were especially interested in observing how the two therapists related and interacted as co-therapists with each other and with the children.

Co-therapy is very frequent in group work (Roller and Nelson, 1996), with advantages and benefits as follows: (a) co-therapy expands creativity and the range of interventions and techniques and so improves transfer and control of countertransference; (b) the therapists complement each other with their knowledge, skills and personalities, while still being able to adopt different positions; (c) the therapy process is improved and shortened; (d) there is mutual support and supervision; (e) responsibilities and decision-making are shared; and (f) the interaction between therapists facilitates the outsourcing of covert conflicts and ambivalence. Furthermore, co-therapy is a highly versatile tool that can be used in many ways and in numerous configurations (Kosch and Reiner, 1984; Hoffman and Laub, 2006). From a psychoanalytical perspective, co-therapy has been focused on pair therapy (Sommantico, 2016). In work with children and adolescents, however, and specifically in the ASD-Sibs setting, there is a research vacuum.

The Society of Group Psychology and Group Psychotherapy provides some arguments against psychotherapy groups conducted with a single therapist (Breeskin, 2013): (a) lone therapists, no matter their expertise, will likely fail to keep up with the richness of the group experience, expressed in non-verbal signals and parallel conversations, which are important details that are at risk of being lost; (b) lone therapists could fail to keep pace with the group’s needs, harming themselves and the participants; and (c) lone therapists in charge of a group means offering professional practice without minimum reference values for the same participants. In short, a therapy partnership offers the opportunity to interpersonally shape a powerful model for the group of participants. A study of 54 co-therapy pairings found that predictors of satisfaction were aspects such as theoretical compatibility and differences in confrontation styles; also significant was being able to select the experience of working together in co-therapy (Bridbord and DeLucia-Waack, 2011). From our perspective, because of the complexity of the interactions, the group phenomenon and the co-therapy relationship, we focused our study on communication, specifically on the interventions and complementarity of the co-therapy relationship.

The training of the therapists is the solid foundation that ensures the studied group become therapeutic. Since the group in question is not a self-help group or a group with a specific requirement, but rather resembles a parent or family group, we may define it as a support group. Support groups, in contrast with *ad hoc* crisis intervention groups, are designed to offer emotional support to persons sharing a common problem or handicap (Scheidlinger, 2005). But unlike a standard support group working on the subject that links it, our intervention group reinforces individual and group work so that the therapeutic work is self-validated.

Also relevant here is Scheidlinger’s idea of the mother-group, which refers to an aspect of identification with the group entity that connotes a covert wish of group members to restore a state of unconflicted well-being, characteristic of an earlier tie to the mother (Scheidlinger, 1974). This longing for a return to that relationship and its unequivocally positive need-gratifying elements is brought directly to bear in and by the group.

The main objective of our research was to study the interaction of therapists and children using a mixed methods framework, an approach that has acquired a certain tradition in recent years (Arias-Pujol and Anguera, 2017; Del Giacco et al., 2019), in accordance with the Guidelines for Reporting Evaluations Based on Observational Methodology (Portell et al., 2015). Specifically, we wanted to identify the existence of possible patterns of behavior in the communicative interactions between children and therapists (a) in turn-taking and (b) breaking down interventions involving clarification (I1), confrontation (I2), and interpretation (I3) for therapist 1 (T1) and therapist 2 (T2) separately and for the two therapists as a single focal subject (co-therapists, CT).

## Materials and Methods

### Design

The observational methodology offers eight types of observational designs (Anguera et al., 2011; Sánchez-Algarra and Anguera, 2013; Anguera and Hernández, 2015) based on three criteria: the number of participants (idiographic or nomothetic), the continuity of the recording (one moment or follow-up) and the number of criteria observed (unidimensional or multidimensional). The design of our research was N/F/M, i.e., it was nomothetic (N) because we studied six subjects (two therapists and four children), it consisted of follow-up (F) because we transcribed six consecutive and multidimensional group sessions, and it was multidimensional (M) because we coded different dimensions of the observed behaviors with concurrent and event-base data from quadrant II of the systematic observation design.

### Participants

The six participants were two women therapists (T1, T2) and four children aged 6–9 years old (P1, P2, P3, P4).

The therapists are clinical psychologists with decades of experience with groups and with autism from a psychoanalytical perspective, and T1, who is older than T2, is also a psychoanalyst.

Regarding the participating children, inclusion criteria were (a) age 6–9 years, (b) attendance at a standard school, and (c) having an ASD-Sib. Exclusion criteria were a diagnosis of pervasive developmental disorder (PDD) and attendance at psychotherapy. The therapists requested the permission of the parents to include their children in the group.

### Intervention Design

The support group intervention was offered in Carrilet Education and Therapy Center (Barcelona, Spain), which specializes in the care of people with autism and their families. A sibling support group offers siblings a space where they can express their feelings and talk about issues in their relationship with the ADS-Sib without fear of hurting their parents. The group has both an educational and therapeutic focus. Autism is discussed with other children who are living a similar experience and with adults who are not members of the family. In words and through play, using plasticine, drawings, etc., the children express feelings, including fear, jealousy, anger, guilt, etc. The psychoanalytical therapists legitimize the ambivalence of the children’s feelings and help them understand and contain their emotions. Through conversation and play these children share feelings that could easily be silenced within the family and this helps them develop their own differentiated identity (Fieschi et al., 2011; Venturella et al., 2014).

The setting is a 1-h monthly meeting over 2 years. Before each session, the therapists send a reminder letter to the children’s home regarding the upcoming session. As material, the group uses two shared boxes, one with colored plasticine and the other with two foldable wooden figures of families (father, mother, girl, and boy), paper, colored crayons and pencils, rubbers, scissors, and a folder for drawings. The group’s activities focus mostly on the wooden dolls and the plasticine, and the plasticine figures made by the children are kept, with some becoming like persons in the group.

At the family level, there are three meetings with parents, two in groups (one each before and after the main sessions) and an individual meeting (Fieschi et al., 2011; Venturella et al., 2014).

In accordance with the principles of the Declaration of Helsinki and the Ethical Code of the General Council of the Official College of Psychologists of Spain, the participants were informed that they were being filmed and parents signed a written informed consent, authorizing the participation of their children in this research. In relation to this study, on the first day of therapy, the reason for filming was explained to the subjects, along with the privacy and confidentiality conditions regarding session content. During the sessions, an observer (a psychologist in training) sat at a distance from the table where the group’s conversation was taking place and took notes.

### Instruments

We used both observation and recording instruments.

Our *ad hoc* observation instrument, following observational methodology canons (Sánchez-Algarra and Anguera, 2013; Portell et al., 2015), combined field format with category systems (Anguera and Izquierdo, 2006; Anguera et al., 2007) and was structured according to the dimensions identified from the conceptual framework. A system of categories was built from the dimensions, which was hierarchical in some of the dimensions (i.e., macro-categories that unfolded into categories). This instrument was designed to fulfill exhaustiveness and mutual exclusivity requirements for each of the category systems. For this reason, all verbal/vocal expressions by the children and the therapists were first transcribed in full. Their analysis resulted in an instrument of 27 codes in four dimensions: (1) turn-taking, (2) group, (3) ASD-Sib, and (4) play. The turn-taking dimension, which reflected turn-taking in speaking, was divided into two macro-categories: therapists and children. The group dimension, which considered the participatory interactions in each turn, was distributed in five macro-categories: body, sound, brief, relationship, and intervention. The ASD-Sibs dimension – the common element among the participating children – reflected all comments regarding the sibling with autism. Finally, play reflected the techniques used to foster interaction and expression within the group.

**FIGURE 2:**
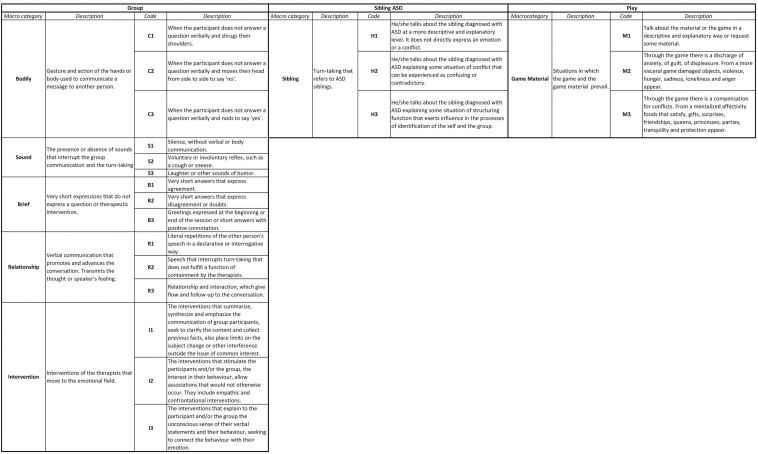
Code description.

Figure 2 depicts the observation instrument with the dimensions along with a description of the macro-categories and codes (the number of the codes does not reflect range or quantity).

**FIGURE 3:**
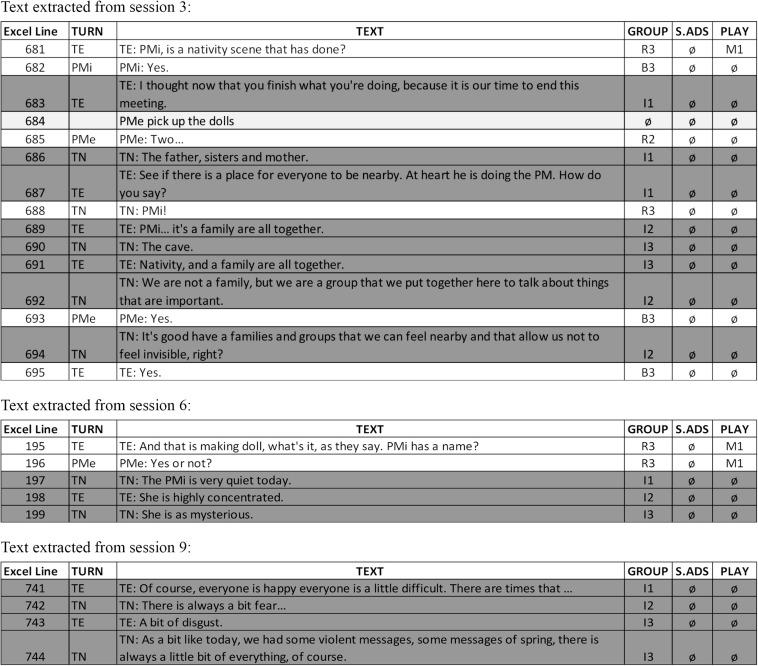
Transcription examples.

We focused on the intervention macro-category (the group dimension) and on specific communications between therapists in relation to the emotional field of the children. To better exemplify the data analyzed below, Figure 3 shows fragments of text that could potentially represent the intervention macro-category (indicated in dark gray).

Used as the recording instrument – to ensure maximum accuracy in data collection – was a video camera. To minimize the reactivity bias of the participants, the camera was positioned discreetly at a high viewing angle in the room.

### Procedure

For the purposes of this study, we used recordings of six sessions from the first year with the group, but excluded three of these as not being fully audible.

All sessions were video-recorded and subsequently transcribed in full. For the transcribed conversations between the children and the therapists, intervention turns were considered as the units of analysis, which were assigned the codes reflecting each dimension from the observation instrument.

### Data Quality Control: Interobserver Agreement

Data quality control was implemented to ensure that codes were correctly assigned. Three observers, previously trained using the approach described by Anguera (2003), analyzed and coded two of the sessions.

Interobserver agreement was measured using Cohen’s kappa (k) (Cohen, 1960, 1968), following Bakeman and Quera (1996, 2011), resulting in values of 90–97% (rated as “very good agreement”). To discriminate interrelationships between the different observers and their standard errors, canonical concordance with a third observer was calculated (Anguera, 1997, 2003; Krippendorff, 2004, 2013), resulting in a Krippendorf’s alpha (α) value of 96% (with values above 80% indicating reliable data). These values indicate that the categories were well defined and had good consistency, with the fact that the system was highly concordant guaranteeing the reliability of the material encoded for subsequent analysis.

### Data Analysis

Since our goal was to detect the existence of possible patterns of behavior in communicative interactions between the therapists and the children, we used intersessional sequential analysis, considered to be the most suitable data analysis technique for our purposes. The sequential analysis technique, developed by Bakeman (1978) and Sackett (1978, 1979) more than 40 years ago, essentially detects whether certain stable behavioral patterns have a greater probability of occurrence than would be expected by chance (Bakeman, 1978; Bakeman and Gottman, 1989; Bakeman and Quera, 2011). Since sequential analysis detects hidden patterns, it is considered an excellent methodology for studying communication in psychotherapy research groups. It has proven to be especially suitable for studying changes that occur over sessions (Arias-Pujol and Anguera, 2004, 2005, 2017; Vaimberg, 2010, 2012; Arias-Pujol, 2011; Roustan et al., 2013; Del Giacco et al., 2019), as well as in families (Gimeno et al., 2006), education (Rodríguez-Dorta and Borges, 2017; García-Fariña et al., 2018), and sports (Lapresa et al., 2013).

In our study we applied it to an analysis of concurrent and event-based quadrant II data. For our analysis, we used GSEQ v.5.1 software (Bakeman and Quera, 2011), in which an algorithm compares the unconditional and conditional probabilities of behavioral occurrences (in our case, prospectively and retrospectively) in the form of frequencies of transition to a criterion behavior, established according to the objectives of the study.

Since the study refers to communication processes, in an initial analysis, we separately considered the intervention turns of the therapists (T1 and T2) and of the grouped children (children) as the criterion and conditional behaviors. In a second analysis, we separately considered the criterion behavior of each therapist for the three forms of intervention, i.e., clarification (T1I1, T2I1), confrontation (T1I2, T2I2) and interpretation (T1I3, T2I3), and used the remaining codes as the conditional behaviors. In a third analysis, the co-therapy (CT) macro-category was taken as the criterion behavior for the three intervention forms (CTI1, CTI2, CTI3) and the remaining codes were taken as the conditional behaviors.

Using the binomial test and the Allison-Liker correction (Allison and Liker, 1982), residuals in lags adjusted from −2 to +2 were calculated [Table 1 shows an example of the adjusted values (RSAJ)]. The analyses were done separately for each of the ten criterion behaviors. Using the GSEQ software, data were entered as .SDS files using the multievent option and then compiled to obtain the .MDS files proposed via the respective. GSQ files were the criterion and conditional behaviors for each analysis and the corresponding lags. The results in .OUT files for each analysis pointed to the existence of various excitatory behavior patterns (>1.96, for α = 0.05) (Bakeman and Quera, 1996, 2011).

**TABLE 1:** Example of adjusted values (RSAJ) obtained for turn-taking between T1, T2 and the child subjects.

**Given:**	**T1**	**T2**	**P1**	**P2**	**P3**	**P4**
**−1**						
T1	–21.58	7.15	6.29	6.61	2.24	4.95
T2	9.72	–28.75	12.24	2.82	7.83	4.97
P1	2.61	15.15	–14.46	–4.35	–5.58	0.2
P2	6.09	2.72	–3.43	–1.64	–2.66	–4.91
P3	4.83	8.91	–7.02	–3.34	–3.82	–5.58
P4	3.7	4.01	–0.07	–3.98	–2.51	–6.13
**0**						
T1	77.78	–31.29	–21.75	–11.3	–14.25	–14.94
T2	–31.29	77.78	–25.14	–13.06	–16.47	–17.27
P1	–21.75	–25.14	77.78	–9.08	–11.45	–12.01
P2	–11.3	–13.06	–9.08	77.78	–5.95	–6.24
P3	–14.25	–16.47	–11.45	–5.95	77.78	–7.86
P4	–14.94	–17.27	–12.01	–6.24	–7.86	77.78
**1**						
T1	–21.58	9.72	2.61	6.09	4.83	3.7
T2	7.15	–28.75	15.15	2.72	8.91	4.01
P1	6.29	12.24	–14.46	–3.43	–7.02	–0.07
P2	6.61	2.82	–4.35	–1.64	–3.34	–3.98
P3	2.24	7.83	–5.58	–2.66	–3.82	–2.51
P4	4.95	4.97	0.2	–4.91	–5.58	–6.13

**FIGURE 4:**
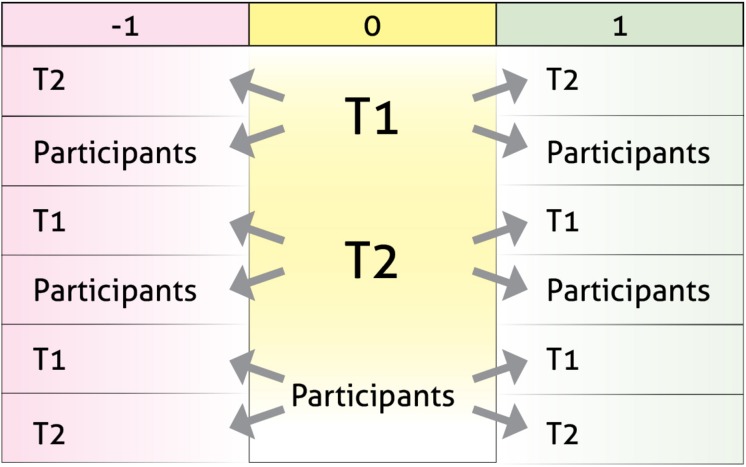
Results for significant behavior patterns corresponding to turn-taking by therapists T1 and T2 and child participants P1, P2, P3, and P4.

*See Figure 4 for a pattern formed from significant results, with values >1.96, for α = 0.05.*

## Results

Our results are described in four sections: the first reflects turn-taking between the therapists and the children, while the remaining three reflect behavior patterns in relation to use of clarification, confrontation, and interpretation by each therapist separately (T1 and T2) and then together in co-therapy (CT). The analysis yielded ten distinct interactive behavior patterns between lags −1 and +1 responding to the question: what precedes and what succeeds therapeutic interventions?

### Relationships Between Separate Turn-Taking by T1 and T2 and the Children as a Group

This first set of patterns with arrows, as shown in Figure 4, point to clear symmetry and reciprocity between T1, T2 and the children. This suggests that communication in the group is fluid and that each person is stimulated by the others to participate.

### Relationships Between T1 Turn-Taking Interventions Using Clarification, Confrontation, and Interpretation and T2, P1, P2, P3, and P4 Turn-Taking

**FIGURE 5:**
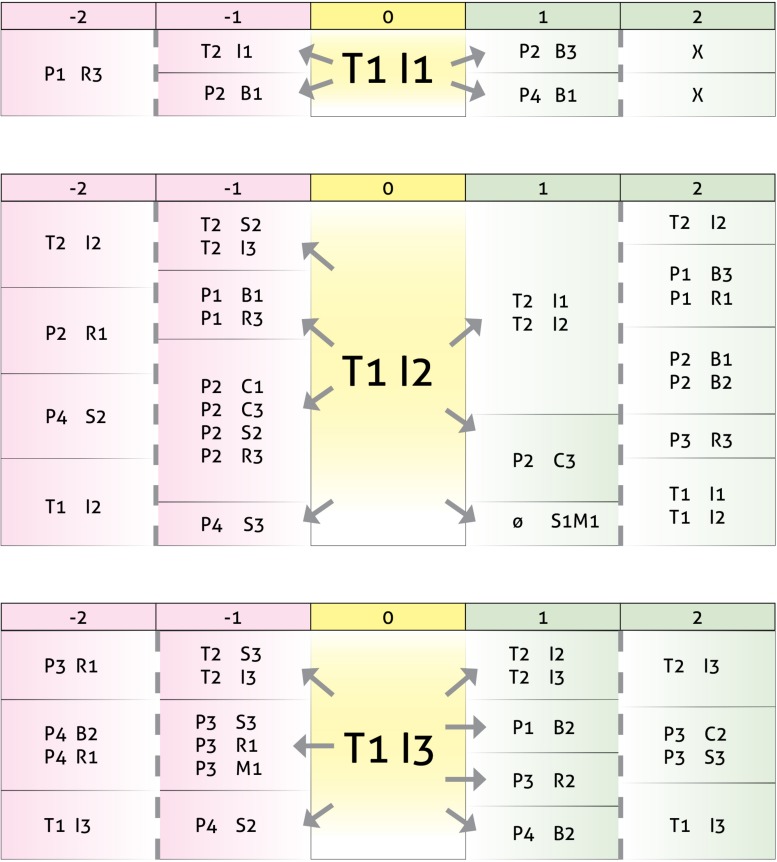
Results in the form of behavior patterns for interactions between turn-taking interventions by therapist T1 as the focal subject, using clarification (I1), confrontation (I2), and interpretation (I3), and turn-taking by therapist T2 and child participants P1, P2, P3, and P4.

The second set of results, depicted in Figure 5, points to differing behavior patterns. In the first pattern, we see how the clarification intervention by T1 arises after the same kind of intervention by T2 or after a brief response by one of the children (P2 B1). These interventions generate short responses (B1, B3) by two other children (P2, P4).

The use pattern of confrontation by T1 is this time much more complex. T1 confrontations follow T2 interpretation interventions (T2 I3) or involuntary behaviors, such as a cough or sneeze (T2 S2), and lead to clarification (I1) and confrontation (I2) interventions by T2. Regarding the children, we see that P1 stimulates T1 with brief interventions (B1) or follow-ups to the conversation (R3), P2 uses non-verbal resources (C1, C3, and S2) and verbal follow-ups to the conversation (R3), whereas P4 laughs. After the therapist has intervened, one of the children (P2) responds with non-verbal approval gestures (C3). The pattern also reflects the possibility of a response in the form of silence (S1) or of a comment regarding the game (M1).

In relation to T1 interpretations, we also see that these follow interpretation interventions (I3) or laughs (S3) by T2 and, in turn, generate confrontation (I2) and interpretation (I3) interventions. As for the children, of note is humor (S3), short answers (R1) and comments regarding the game (M1) by P3 or involuntary reflexes, such as coughs or sneezes (S2) by P4 prior to the interpretation by T1. *A posteriori*, the interpretation generates brief expressions of disagreement or doubt (B2) in two of the children (P1 and P4) and interruptions (R2) by another child (P3).

**FIGURE 6:**
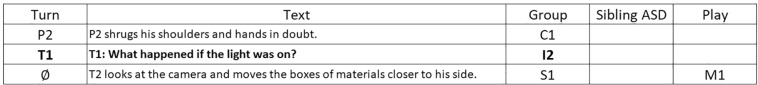
T1 – Significant pattern example.

Figure 6 shows an example of a communicative behavior pattern in the use of confrontation by T1.

### Relationships Between T2 Turn-Taking Interventions Using Clarification, Confrontation, and Interpretation and T2, P1, P2, P3, and P4 Turn-Taking

**FIGURE 7:**
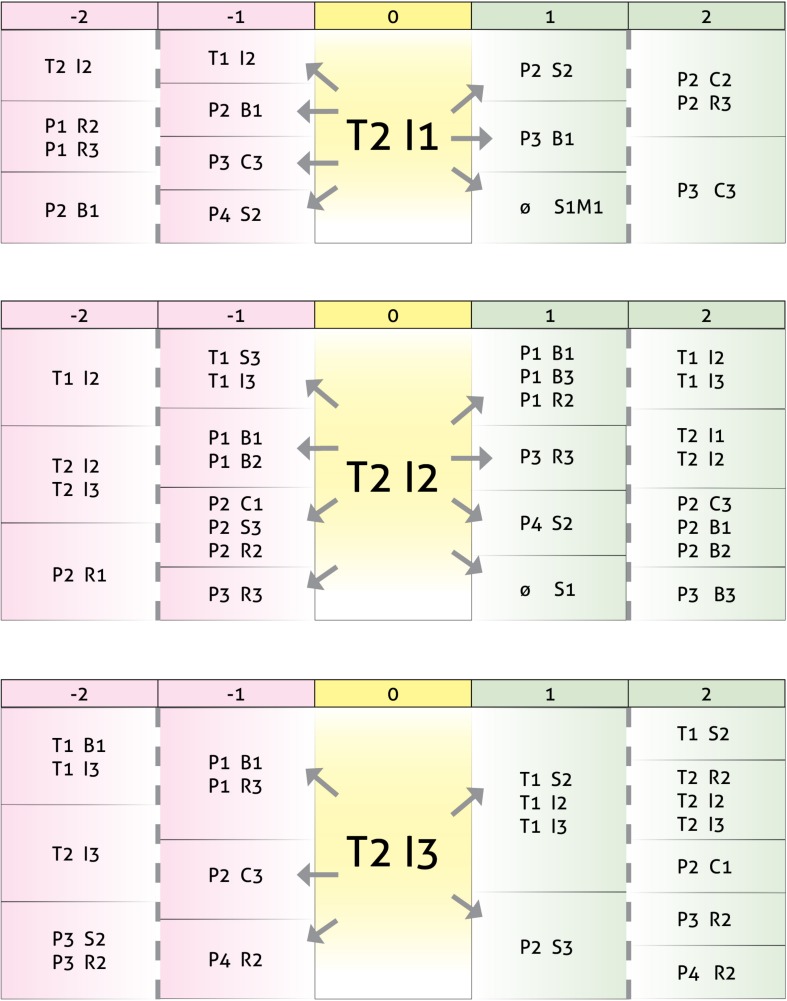
Results in the form of behavior patterns for interactions between turn-taking interventions by therapist T2 as the focal subject, using clarification (I1), confrontation (I2), and interpretation (I3), and turn-taking by therapist T1 and child participants P1, P2, P3, and P4.

This third set of results, depicted in Figure 7, is even more complex that the previous set. In the first pattern we see how the clarification intervention (I1) by T2 follows the clarification intervention (I2) by T1 or a brief response (B1), non-verbal response (C3) or coughing or sneezing (S2) by the children (P2, P3, P4, respectively). T2 clarification interventions generate coughing or sneezing (P2 S2), very short responses or verbal agreement (P3 B1), silence, or comments regarding the game.

As for confrontations (I2) by T2, these follow interpretation or laughter by T1. *A priori* of the confrontation intervention, the children (P1) briefly express agreement (B1) or doubt (B2), or respond non-verbally, defensively or with laughter (P2), or express their collaboration with the conversation (P3). The confrontational interventions by T2 generate short responses, positive answers or interruptions by P1, collaborative responses by P3, coughing or sneezing by P4 or silence in the whole group.

In relation to the children, the interpretations of T2 arise from brief expressions of gratitude or collaborative interventions by one child (P1), interruptions by another child (P4) and a non-verbal response by yet another child that stimulates laughter (P2). The interpretations of T2 stimulate interpretation (I3) or confrontation (I2) interventions, and also coughing or sneezing (S2) by T1 and laughter or other sounds reflecting humor in P2.

**FIGURE 8:**
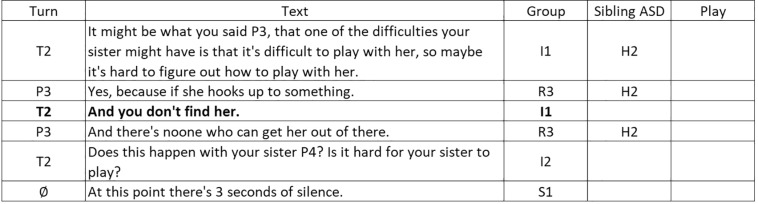
T2 – Significant pattern example.

Figure 8 shows an example of a communicative behavior pattern in the use of clarification by T2.

### Relationships Between CT Turn-Taking Interventions Using Clarification, Confrontation, and Interpretation and P1, P2, P3, and P4 Turn-Taking

**FIGURE 9:**
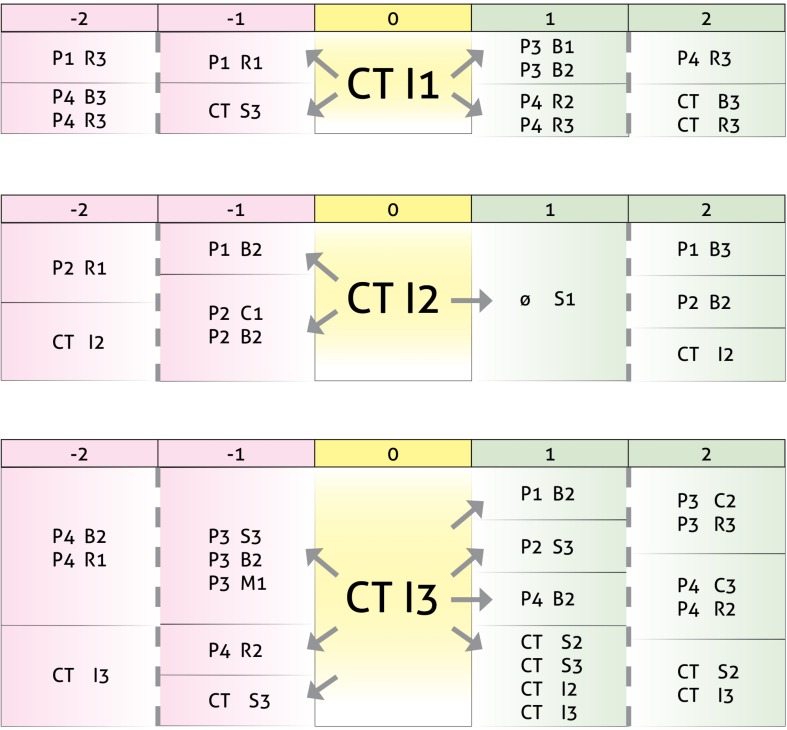
Results in the form of behavior patterns for interactions between turn-taking interventions by the therapists together (CT) as the focal subject, using clarification (I1), confrontation (I2), and interpretation (I3), and turn-taking by child participants P1, P2, P3, and P4.

This final set of results shows that when T1 and T2 are grouped together (i.e., CT), communicative patterns are simplified somewhat, as shown in Figure 9. The first detected pattern is clarification interventions following repeated questions by one child (P1 R1) or laughter or other sounds reflecting humor by the therapists, generating brief assent (B1) or doubt (B2) interventions in another child (P3) or interruptions (R2) or interventions that foster progress (R3) in yet another child (P4).

When the focal subject is CT, confrontation interventions arise after non-verbal responses by P2 or brief interventions expressing doubt or disagreement by two of the children (P1, P2), generating silence in the group.

Finally, CT interpretation interventions arise after laughter or other sounds reflecting humor in one of the children or the therapists (P3, CT), or brief interventions expressing doubt or disagreement (B2) by one of the children (P3) or interruptions (by P4). These interpretations generate other brief interventions expressing doubt or disagreement (P1, P4), laughter (P2, CT) or other confrontation interventions by the therapists (CT).

**FIGURE 10:**
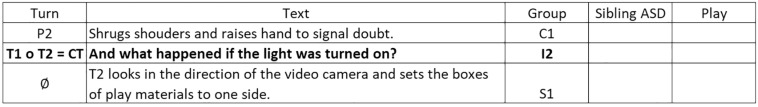
CT – Significant pattern example.

Figure 10 shows an example of a communicative behavior pattern in CT use of confrontation.

## Discussion

The intervention macro-category was designed as a resource and as a means of communication for psychoanalytical therapists T1 and T2. Unlike the other categories, interventions introduce new variables and new emotional experiences and help to develop new mental models. The changes seen in the children were the result of the attitudes and verbal interventions of the therapists, whether clarification (I1), confrontation (I2), or interpretation (I3).

In the group, both therapists perform clarification after a brief communication (body or sound). As the therapists do not introduce feelings or ideas that the children have not expressed, these continue with a similar discourse in the form of a brief response, some sound or silence, followed by manipulation of the material. Given its simplicity and neutrality, this intervention generates confidence and helps improve connections between participants (Coderch, 2009).

Confrontation arises when thoughts are explored in depth (Ferro and Civitarese, 2016). In the group, this type of intervention occurs after brief, body and sound responses, as well as after responses of a more relational nature. Since the therapist highlights omitted aspects, the response is mostly silence, in some cases accompanied by manipulation of the material, by a brief communication (body or sound) and, occasionally, by a more relational tracking response. To a lesser or greater extent, the response is directive. The fact that the main objective may be to facilitate the transition from clarification to interpretation would explain a downward tendency during the group session.

Confrontation helps children overcome their difficulties in expressing themselves. It also facilitates clarification and interpretation interventions (Lichtenberg, 2016). Interpretation is a basic psychoanalytic instrument. To interpret is to explain the unconscious meaning of statements to patients. In the group, interpretation interventions are also represented after brief, body, sound, and relational communications. However, they add to the sequence of material and play, through which therapists inform children of unconscious mental processes that direct and condition their relationships with others. Subsequent responses are usually given by the therapists themselves or, briefly, by the children.

The therapist’s efforts focus on bringing the patient to an understanding of how to balance their inner fantasies with influences from the external world.

The emergence of behavioral patterns of silence or of responses that reflect collaboration or dissatisfaction in the children in response to the interventions of the therapists is consistent with results obtained in previous research on the role of the psychoanalytic therapist in group sessions (Arias-Pujol and Anguera, 2004, 2005) and in individual sessions (Arias-Pujol et al., 2015).

In our sequential analysis it was found that T1 activates clarification and confrontation interventions by T2 but does not follow up on these interventions. The opposite happens in the interpretation interventions, where T1 does not activate T2 interventions, but does follow up. T2 activates all the three types of interventions by T1 and follows up on confrontation and interpretation interventions.

Both therapists are women. T1 (the older therapist) seems to assume a greater role in containment, tolerance, and follow-up. The younger T2 seems to play a role that is more activating, verbal and available (Kosch and Reiner, 1984). As noted in the results, therapeutic interventions present significant sequences in the response patterns that precede and succeed them.

The co-therapy (CT) analysis, more global than the analysis of individual relationships within the group, is characterized by more general aspects and issues reflecting the group as a whole. Along these lines, it can be observed that in the CT clarification intervention, P1 is hidden in previous analyses of this category, and, at the same time, the leadership of the other children is obscured. While the subsequent responses are similar to those of the therapists in isolation, there is no room for the silence represented above.

In the confrontation, the two protagonists of separate interventions by the therapists stand out, but therapists conceal what the other children express and leave silence evident. In this case, if the game is not followed up, then this is the only possible response.

Interpretation implies deeper intervention. The co-therapeutic result helps protect the children, since previous and subsequent follow-ups to the interpretation intervention take place between the therapists themselves (Blum, 2016). Thus, T2 activates T1’s interpretation and follows up, and T1 activates T2’s interpretation and follows up.

These functions do not follow a rule, nor are they permanent. The therapists adapt them to the requirements of the children and complement them in their interventions. Noteworthy is the sum of the attitudes of T1 and T2 in their co-therapeutic work. The fact that they share a theoretical framework and have experience of working together boosts their expertise in creating a facilitating space and in allowing interactions through dialog and play (Bridbord and DeLucia-Waack, 2011). However, we consider that one of the most valuable aspects of this kind of group is the possibility of representing a certain “family model,” where therapists are representatives of adults and of children as siblings. Scheidlinger (1974) specifies the need for group members to establish wellbeing regarding the mother (therapists), as a powerful force of identification and connection for the group as a whole.

Of course, there is no differentiation of functions other than those determined by the personal and professional characteristics of each therapist; however, alternating between different functions means they participate in the transference process.

As observed in our results, analyzing the profiles of the therapeutic partners (co-therapists) draws attention to children and responses not observed in the individual analyses of each therapist’s interventions.

The results also show that children speak little of their siblings with autism and participate in the sessions spontaneously with all kinds of interventions (liking, disliking, laughter, play, etc.). The communicative richness evident in their behavior patterns reinforces the importance of offering this type of intervention for children with ASD-Sibs (Shivers et al., 2018). The support group helps them think about and of themselves and facilitates their development of a differentiated identity (Fieschi et al., 2011; Centre Educatiu i Terapèutic Carrilet, Alcácer et al., 2013; Venturella et al., 2014).

## Conclusion

In addition to our sequential study of the interventions, we were able to observe parallels and interwoven relationships for the two therapists, who complement each other in the direction in which their interventions are intended. Basic aspects of co-therapy include shared impressions, continuous exchanges and integrated countertransference aspects (Cabré, 2002). As noted by Scheidlinger (2005), most group therapists tend to adhere to a pluralistic-integrative orientation that appears to be suited to the complexity of individual and group-level manifestations.

A limitation of our study is the fact that results cannot be generalized, given the small size of our sample, both in terms of the number of sessions and the variety of attitudes. Rather, the group should be considered as a unique case analyzed in depth in terms of individual processes.

The methodological characteristics used are highly appropriate for a study of group processes and human interactions (Anguera and Hernández, 2015). However, the complexity and diversity of behaviors to be observed meant we were unable to use a standard instrument. Therefore, a great deal of time was devoted to preparing a tailormade instrument, based exclusively on the profile of the studied group. Our experience suggests that, as proposed by Breeskin (2013), it would be of great interest to broaden the theoretical foundations of co-therapy, as, with further monitoring and evaluation, other groups may benefit from the developed *ad hoc* instrument and so evolve to new lines of research.

## Ethics Statement

This study was carried out in accordance with the recommendations of “Comitè d’Ètica de la Recerca (CER-URL)” with written informed consent from all subjects. All subjects gave written informed consent in accordance with the Declaration of Helsinki. The protocol was approved by the “Comitè d’Ètica de la Recerca (CER-URL).”

## Author Contributions

MV developed the project and performed the sequential analysis. VC and XC contributed to the documenting and writing of the manuscript. EA-P supervised the project, designed the study, developed the methodology, and drafted the manuscript.

## Conflict of Interest Statement

The authors declare that the research was conducted in the absence of any commercial or financial relationships that could be construed as a potential conflict of interest.
